# Live Imaging of Mitosomes and Hydrogenosomes by HaloTag Technology

**DOI:** 10.1371/journal.pone.0036314

**Published:** 2012-04-27

**Authors:** Eva Martincová, Luboš Voleman, Vladimíra Najdrová, Maximiliano De Napoli, Shiri Eshar, Melisa Gualdron, Christine S. Hopp, David E. Sanin, Dumizulu L. Tembo, Daria Van Tyne, Dawn Walker, Michaela Marcinčiková, Jan Tachezy, Pavel Doležal

**Affiliations:** 1 Department of Parasitology, Faculty of Science, Charles University, Prague, Czech Republic; 2 Laboratorio de Parasitología Molecular, IIB-INTECH, CONICET-UNSAM, Chascomús, Provincia de Buenos Aires, Argentina; 3 Department of Microbiology and Molecular Genetics, The Kuvin Center for the Study of Infectious and Tropical Diseases, IMRIC, The Hebrew University-Hadassah Medical School, Jerusalem, Israel; 4 Christian De Duve Institute of Cellular Pathology, Brussels, Belgium; 5 Malaria Centre, London School of Hygiene & Tropical Medicine, London, United Kingdom; 6 Department of Biology, University of York, York, United Kingdom; 7 Malawi-Liverpool Wellcome Trust Clinical Research Programme, Chichiri, Blantyre, Malawi; 8 Department of Immunology and Infectious Diseases, Harvard School of Public Health, Boston, Massachusetts, United States of America; 9 Integrated Biomedical Sciences Center for Microbial Interface Biology Department of Internal Medicine, Columbus, Ohio, United States of America; University of Melbourne, Australia

## Abstract

Hydrogenosomes and mitosomes represent remarkable mitochondrial adaptations in the anaerobic parasitic protists such as *Trichomonas vaginalis* and *Giardia intestinalis*, respectively. In order to provide a tool to study these organelles in the live cells, the HaloTag was fused to *G. intestinalis* IscU and *T. vaginalis* frataxin and expressed in the mitosomes and hydrogenosomes, respectively. The incubation of the parasites with the fluorescent Halo-ligand resulted in highly specific organellar labeling, allowing live imaging of the organelles. With the array of available ligands the HaloTag technology offers a new tool to study the dynamics of mitochondria-related compartments as well as other cellular components in these intriguing unicellular eukaryotes.

## Introduction

In recent years studies of anaerobic protists such as *Giardia intestinalis* and *Trichomonas vaginalis* have revealed a number of exciting aspects of their cell biology, including cytoskeleton structures, vesicular transport and organelle biogenesis [1]–[5]. Besides unique cellular structures [6]–[8], many of the common eukaryotic processes have been stripped to their essentials in these protists e.g. [9], [10]. The combination of their parasitic lifestyle, anaerobic metabolism and their evolutionary position [11] makes them attractive objects to study.

One of the features typical to anaerobic protists is the absence of ‘classical’ mitochondria, herein represented by organelles called mitosomes in *G. intestinalis* and hydrogenosomes in *T. vaginalis* [12]. Mitosomes, the simplest mitochondria-related compartments, seem to have lost all but the single pathway of iron-sulfur cluster assembly [4]. Compared to mitosomes, hydrogenosomes are more elaborate organelles, possessing substrate level ATP synthesis as well as iron and amino acid metabolism [13], [14]. Moreover, recent proteomic studies of hydrogenosomes suggest that many more pathways are yet to be described [10], [15].

Characterization of cellular organelles and their dynamics strongly relies on the concerted action of reverse genetics and live cell imaging. While particular advancements have been achieved in the former (e.g. gene silencing and protein overexpression) [16]–[20], only limited technical innovations have been introduced into the latter [21], [22].

GFP and its derivatives are the first choice of reporters for live imaging in aerobic eukaryotes. They offer great protein stability as well as a broad range of spectral variants that enable multichannel studies. However the major drawback for their widespread use in anaerobic protists is the formation of the GFP fluorophore [22], [23]: upon translation and protein folding the fluorophore is formed from the tripeptide Ser 65-Tyr 66-Gly 67 by an intramolecular cyclization, which requires the presence of molecular oxygen [24], [25]. This reaction does not require additional proteins and occurs spontaneously in all eukaryotic compartments, except within anaerobic cells, which employ oxygen scavenging pathways in order to limit its toxic effects [26], [27]. Cells can be temporarily oxygenated and observed under the microscope [7], [28] . While this approach has proven to be efficient for large cellular structures such as the cytoskeleton [21], [29], the organelles like mitosomes and hydrogenosomes exhibit only very weak labeling. Additionally, the double membrane surrounding the organelles may have limited capacity to import GFP.

Alternative approaches for live cell imaging exploit the use of chemical fluorescent tags, which form covalent or noncovalent bonds with the reporter protein or peptide [30]. Of these, SNAP and CLIP tags are commonly used for both extra- and intracellular labeling [31], [32]. The SNAP tag was successfully used to track the distribution of *G. intestinalis* RabA homologue in the live parasite [22]. However, the use of the tag has been limited to this single study so far.

In this work, we decided to test a newly developed tag termed HaloTag, which utilizes a mutant form of haloalkane dehalogenase as a reporter protein. While the original enzyme hydrolyzes alkylhalides into a free halide and a primary alcohol, the H289Q mutant form of the protein (HaloTag) leaves free halide but remains covalently bound to the alkyl chain [33]. Thus, when a ligand with the alkylhalide chain is exposed to the native HaloTag, it is specifically bound by a covalent bond. The lack of dehalogenase activity among eukaryotes guarantees very low unspecific background labeling.

Here, we report the successful introduction of the HaloTag into vectors for stable expression in *G. intestinalis* and *T. vaginalis.* Moreover, using a TMR-halo ligand we were able to show live images of mitochondria-related compartments in these two anaerobic protists for the first time.

## Materials and Methods

### Cell strains

The *G. intestinalis* strain WB (ATCC 30957) was grown in TYI-S-33 medium supplemented with 10% heat-inactivated bovine serum, 0.1% bovine bile, and antibiotics. The *T. vaginalis* strain T1 was grown in TYM pH 6,2 medium supplemented with 10% heat inactivated horse serum. Both organisms were cultured at 37°C.

### Preparation of cell fractions

*G. intestinalis* trophozoites were harvested in ice-cold PBS, washed once in ST buffer (250 mM sucrose, 0.5 mM KCl, 10 mM Tris [pH 7.2]) and suspended in ST buffer with protease inhibitors 50 µg/ml *N-*a-tosyl-L-lysine chloromethyl ketone and 10 µg/ml of leupeptin. Cells were lysed on ice using sonication, during which the cell integrity was checked under the microscope. The lysate was centrifuged twice at 2450× g for 10 minutes to remove unbroken cells, nuclei and residual cytoskeleton. Supernatant was transferred to a new tube and the centrifugation step repeated twice. The resulting supernatant was spun down at 180 000× g for 30 minutes. Final supernatant and pellet contained the cytosolic and high-speed pellet fraction, respectively.

*T. vaginalis* cells were harvested, washed once in ST buffer and suspended in ST buffer containing protease inhibitors (see above). Cells were sonicated on ice and the lysate was twice centrifuged at 2450× g (see above). Supernatant was spun down at 180 000× g for 30 minutes. The final supernatant corresponded to the cytosolic fraction. The pellet was resuspended in 1 ml of ST buffer, transferred to a new microcentrifuge tube and spun down at 30 000× g for 10 minutes. The Resulting pellet contained a white layer of lysosomes resting on top of a brown pellet of hydrogenosomes. Lysosomes were carefully removed using a pipette and this step was repeated once more. The final pellet corresponded to the hydrogenosomal fraction.

### Cloning and stable cell transformation

#### *G. intestinalis*

First, pTG vector (gift from Francis D. Gillin, [34]) was modified to contain NdeI PstI sites. The polylinker containing EcoRV, NdeI, XhoI, PstI, NsiI, MluI and ApaI sites was introduced into the vector using 5′-CATGGATATCCATATGCTCGAGCTGCAGATGCATACGCGTATGGTGAGCAAGGGCGAGGAG-3′ and 5′-GATCGGGCCCTCACTTGTACAGCTCGTCCAT-3′ primers. The PCR product was digested by EcoRV and ApaI and ligated into EcoRV/ApaI linearized pTG vector. The 300 bp of 5′UTR of *G. intestinalis* ornithine carbamoyl transferase (OCT) DNA sequence was amplified using 5′-CATGGATATCGAATTCGATGCTTCG-3′ and 5′-CATGCATATGTTTAATTTTCAGCCTCTACTG-3′ primers, digested by EcoRV and NdeI primers and ligated into modified pTG vector. The HaloTag DNA sequence was amplified from pHT2 vector (Promega) using 5′-ATGCTGCAGATGGGATCCGAAATCGGTACA-3′and 5′-CATGGGGCCCTTAGCCGGCCAGCCCGGGGAG-3′ oligonucleotides. The resulting PCR product was digested by PstI and ApaI and ligated into modified pTG vector. *G. intestinalis* IscU was amplified from genomic DNA using 5′-CTAGCATATGATGACTTCTGATGCCGCAGAT-3′ and 5′-GACTATGCATAGAAGACTTTGATACCTGTAT-3′ oligonucleotides. The product was digested by NdeI and NsiI and ligated into modified pTG vector containing HaloTag coding sequence.

#### *T. vaginalis*

For expression in *T. vaginalis*, the HaloTag DNA sequence was amplified from pHT2 vector using 5′-CATGAGATCTATGGGATCCGAAATCGGTACA-3′ and 5′-GCTACTCGAGTTAAGCGTAATCTGGAACATCGTATGGGTAGCCGGCCAGCCCGGGGAGCCA-3′. The C-terminal hemagglutinin (HA)-tag was introduced into the construct as a part of the reverse primer. The PCR product was digested by BglII and XhoI and ligated into BamHI/XhoI linearized TagVag2 vector containing a gene encoding hydrogenosomal frataxin. Both organism were electroporated using modified protocols published in [35], [36]. Briefly, three hundred micro liters of *T. vaginalis* and *G. intestinalis* at approximate concentration 2,5×10^8^ cells/ml and 3,3×10^8^ cells/ml, respectively, were electroporated with 50 ug of the plasmid using a Biorad Gene Pulser under the time constant protocol (Tc = 175 ms, U = 350 V). Transfectants were maintained under pressure of selective antibiotics (57 ug/ml of puromycin for *G. intestinalis* and 200 ug/ml for *T. vaginalis*).

### Halo-labeling and immunofluorescence microscopy

Cell were incubated for 30 minutes in regular growth media supplemented with HaloTag TMR Ligand (1: 500 dilution) at 37°C. After the incubation the cells were pelleted at 1500× g and washed twice with fresh media. Cells were then incubated for 60 minutes at 37°C, pelleted and resuspended in fresh media or PBS. For immunofluorescence, the cells were incubated on slides for 15 minutes, fixed in −20°C methanol for 5 minutes and transferred to −20°C acetone for 5 minutes. Blocking and immunolabeling was performed in 0,25% Gelatin, 0,25% BSA, 0,05%.

Tween20 in PBS 7,4 using specific rabbit polyclonal antibodies raised against *T. vaginalis* malic enzyme and *G.intestinalis* Tom40 . Primary antibodies were decorated by Alexa Fluor 488 anti-rabbit antibody. Slides were mounted in hard set Vectashield containing DAPI. For live cell imaging, labeled *G. intestinalis* cells were allowed to attach to the surface of 96 well optical bottom plates and imaged directly. Labeled *T. vaginalis* cells were mounted in low temperature-melting 2% agarose dissolved in PBS and analyzed by microscopy. Cells were observed using an OLYMPUS Cell-R, IX81 microscope system and images processed by Fiji (http://fiji.sc/wiki/index.php/Fiji). During all steps cells were protected from light.

## Results and Discussion

Mitosomes and hydrogenosomes can be found in anaerobic protists from different eukaryotic lineages. Recent phylogenetic and functional data have shown that these double membrane bound organelles represent long evolved mitochondrial forms adjusted to anaerobic environments [12]. While devoid of many typical mitochondrial functions, they contain unique metabolic adaptations as well as simplified versions of intricate molecular processes occurring in mitochondria [37]–[39]. To date only limited information is available on their biogenesis, inheritance and related membrane dynamics [40]. In order to follow these processes in living cells we have introduced HaloTag technology into both *G. intestinalis* and *T. vaginalis.*

The coding sequence of HaloTag was introduced into *G. intestinalis* and *T. vaginalis* episomal vectors pTG and TagVag2, respectively [34], [39] . Transcription from these vectors is driven by promotor regions in 5′ UTRs of highly expressed ornithine carbamoyl transferase and succinyl-CoA thiokinase [34], [41], respectively, which ensure strong constitutive protein expression in both organisms. For specific labeling of mitochondria-related organelles in these anaerobic protists, the HaloTag was inserted as a C-terminal fusion to the mitosomal and hydrogenosomal marker proteins GiIscU and TvFtx, respectively [13], [42].

**Figure 1:**
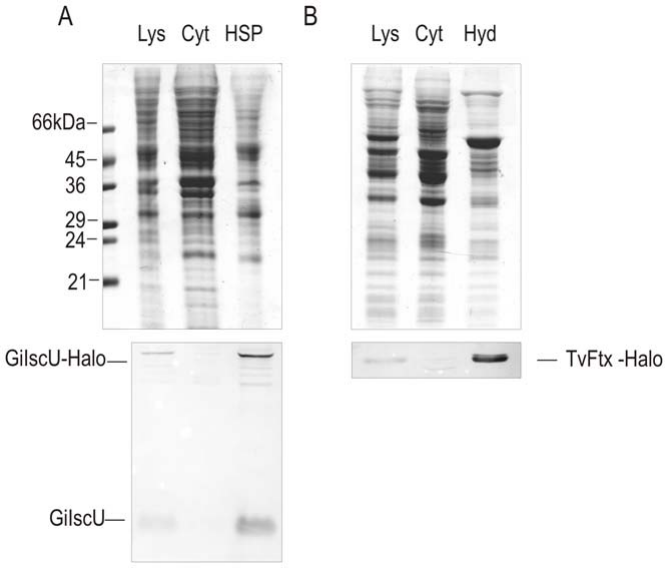
Expression of HaloTagged proteins in *G. intestinalis* and *T. vaginalis*. Western blot analyses of cellular fractions of *G. intestinalis* and *T. vaginalis* transformants expressing GiIscU-Halo and TvFtx-Halo fusions, respectively. A) GiIscU-Halo was detected by specific anti-IscU polyclonal antibodies in cell lysate and high-speed pellet (HSP). Two bands in these fractions represent the nuclear encoded (GiIscU) and episomally encoded HaloTag fusion (GiIscU-Halo). B) TvFtx-Halo product was detected by anti-HA monoclonal antibodies in *T. vaginalis* cellular fractions. The fusion protein was found exclusively in cell lysate and in hydrogenosomes. The upper panels demonstrate the protein profile on the coomassie stained SDS-PAGE gel. Lys-lysate, Cyt-cytosol, HSP-high-speed pellet, Hyd-hydrogenosomes.

Expression of proteins fused to the HaloTag was determined on western blots of cellular fractions (Figure 1). *G. intestinalis* IscU-HaloTag fusion was detected by specific polyclonal antibody raised against mitosomal IscU. Two dominant protein bands of approximately 15 kDa and 50 kDa were detected, which is consistent with the expected molecular weights (the size of HaloTag is 33 kDa) (Figure 1A). While the lower band corresponded to the mature form of nuclear encoded IscU, the upper band represented IscU-HaloTag fusion. The specific signal was present in the lysate and high speed pellet fraction, which is in addition to other vesicular structures enriched for mitosomes. Additional weak protein bands were detected, which likely corresponded to partially proteolytically degraded protein forms.

In order to detect HaloTagged hydrogenosomal frataxin in *T. vaginalis*, an additional single hemagglutinin (HA)-tag was introduced to the C-terminus of the HaloTag sequence. Using anti-HA antibodies the protein band of about 47 kDa, corresponding to the expected protein fusion size, was detected in the cell lysate and hydrogenosomal fractions (Figure 1B).

In both organisms, the HaloTag fusion proteins were expressed at a moderate level with no growth defect obvious in daily culturing, indicating that the tag does not interfere with the cellular metabolism of the anaerobic eukaryotes, similar to what has been shown in mammalian cells [33].

**Figure 2:**
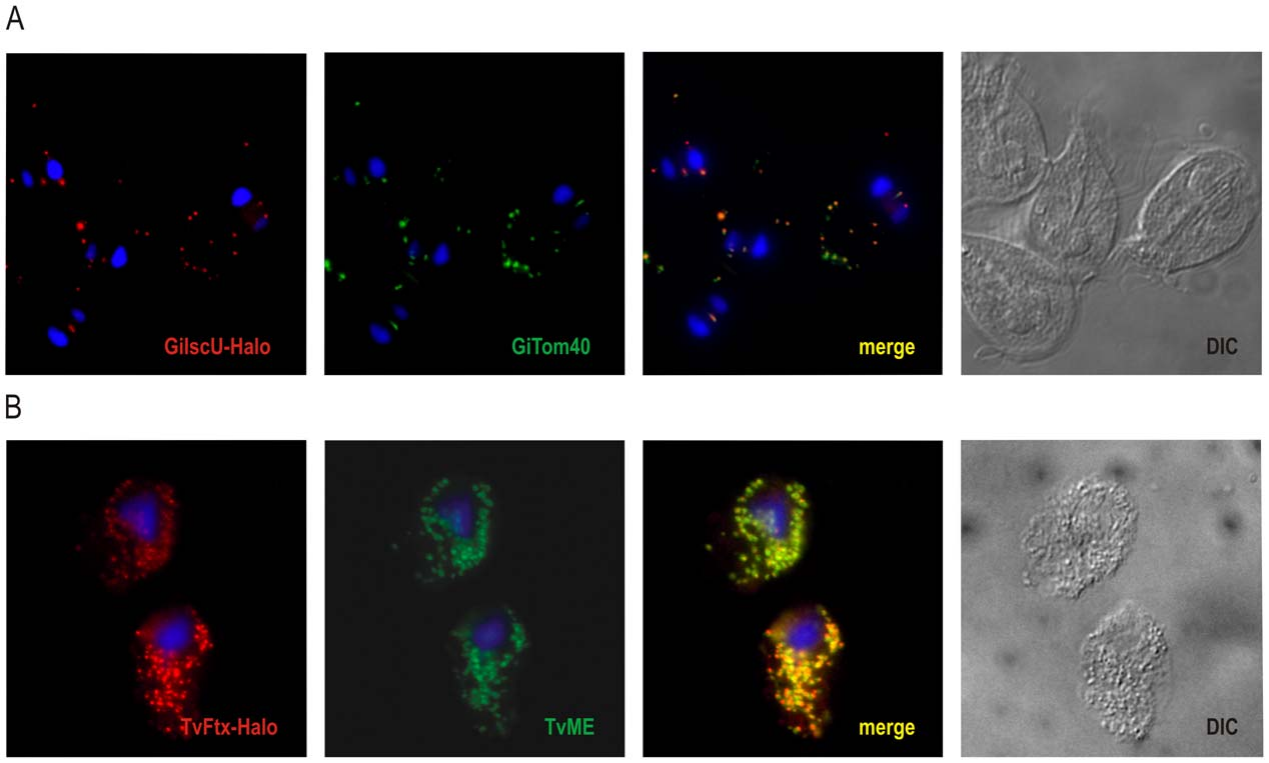
Mitosomal and hydrogenosomal localization of HaloTagged proteins. Immunofluorescence analyses of *G. intestinalis* and *T. vaginalis* transformants expressing GiIscU-Halo and TvFtx-Halo fusion, respectively. Cells were incubated with TMR-Halo ligand (red), washed and fixed for immunofluorescence analysis. A) TMR-Halo labeled *G. intestinalis* cells were fixed and labeled by anti-Tom40 specific polyclonal antibodies (green). B) TMR-Halo labeled *T. vaginalis* cells were fixed and decorated by anti-malic enzyme specific polyclonal antibodies (green). Nuclei were stained with DAPI (blue).

In order to confirm that the fusion protein is targeted to mitochondria-related compartments of *G. intestinalis* and *T. vaginalis*, cells were labeled with HaloTag TMR ligand, fixed and immunolabeled with specific antibodies raised against organellar marker proteins. In *G. intestinalis*, mitosomes were labeled by Tom40-specific antibody [37]. Tom40 is a conserved protein of the outer mitochondrial/mitosomal membrane and its detection revealed typical mitosomal distribution within *G. intestinalis* cells: the central array of mitosomes between the two nuclei as well as the peripheral ones scattered throughout the cytoplasm. The HaloTag signal from GiIscU was found to be in perfect agreement with Tom40, revealing highly specific mitosomal compartment labeling in *G. intestinalis* (Figure 2A).

In contrast to mitosomes, which are scarce, *T. vaginalis* hydrogenosomes are abundant organelles distributed along the major cytoskeletal structures such as the costa and axostyle. Malic enzyme is the most dominant hydrogenosomal protein [43] and its detection in fixed TMR-Halo ligand-labeled cells revealed typical hydrogenosomal distribution. The same pattern was obtained with TMR labeled HaloTag, as indicated in the merged image (Figure 2B).

These experiments showed that the HaloTag TMR ligand is a membrane-permeable ligand in both organisms, capable of diffusing across the cell membrane as well as the two membranes surrounding the mitosomes and hydrogenosomes. Although some background labeling was detected using HaloTag in mammalian cells [31], no such signal was found in two anaerobic organisms used in this study.

**Figure 3:**
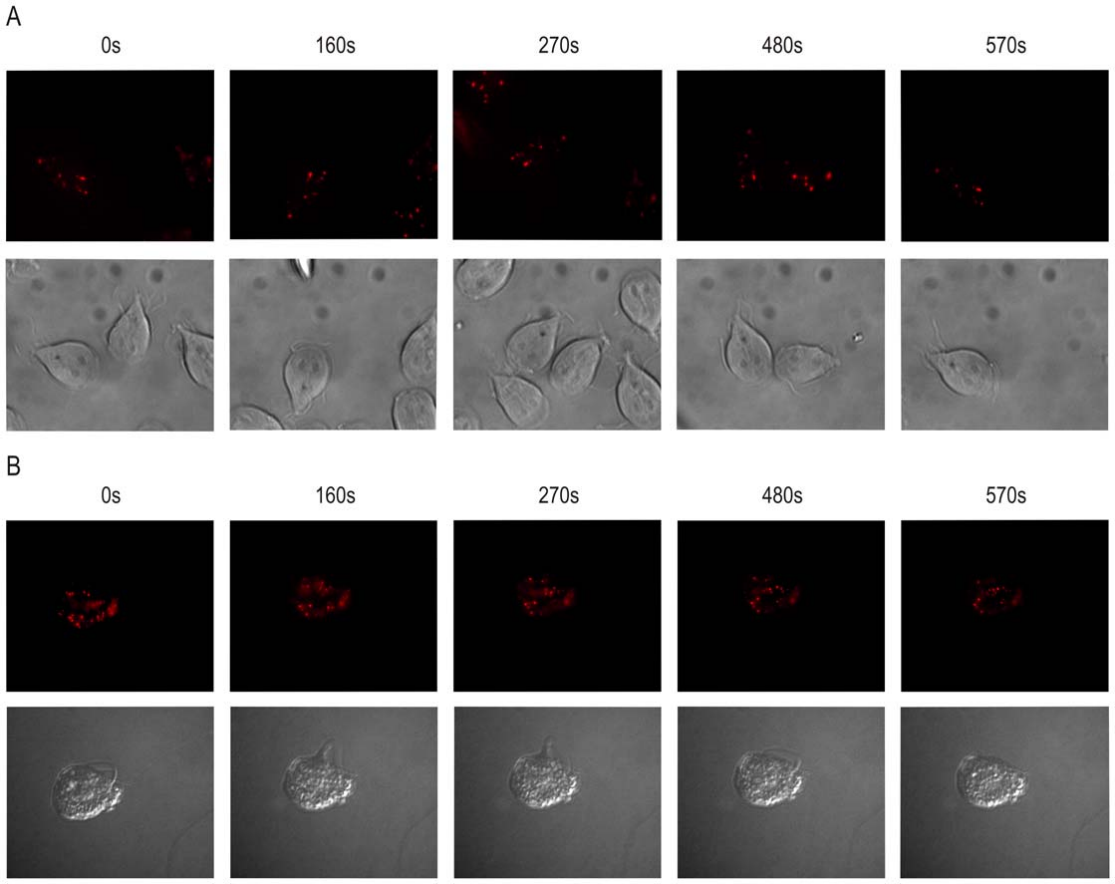
Live imaging of mitosomes and hydrogenosomes. Halo-TMR labeled organelles were followed in living cells. A) Labeled *G. intestinalis* cells were allowed to attach to the bottom of the well and directly observed while B) the labeled *T. vaginalis* cells were mounted in 2% agarose and then submitted to microscopy. Five different snapshots in time are shown. The original movies are part of the supplementary data.

Following the co-localization experiments, labeled cells were observed live for various time periods (Figure 3). While attached *G. intestinalis* trophozoites could be observed directly in optical bottom plates filled with medium (Figure 3A, Supplementary Movie S1 and S2), *T. vaginalis* were mounted in 2% low melting agarose in order to slow down the rapidly moving cells (Figure 3B, Supplementary Movie S3 and S4). In both parasites, the specific fluorescence signal could be followed visually for more than 60 minutes. Notably, for prolonged cell observation an anaerobic chamber would be necessary.

In summary, these experiments demonstrate the applicability of HaloTag in labeling the mitochondria-related organelles of *G. intestinalis* and *T. vaginalis.* These tiny double membrane bound organelles have been some of the most challenging cellular structures for live imaging in anaerobic eukaryotes, and to our knowledge this work is the first report of its kind.

HaloTag technology is relatively new to the cell biology. It exhibits excellent specificity and fast chemistry but as true for other large protein tags such as the fluorescent proteins or SNAP-tag, its major drawback is the size, which may interfere with the function of the carrier protein [30]. When possible the imaging studies rely on GFP and other recently characterized fluorescent proteins e.g. [44], [45]. In these cases, the chemical tags such as HaloTag, SNAP-tag or tetracystein helix motif [46] offer additional customizable labeling, especially suitable for pulse-chase [47] or FRET experiments. In the anaerobic unicellular organisms or the anaerobic tissues of some invertebrates the GFP maturation requires an extra oxygenation step, which may perturb narrow physiological conditions. In these cases, the chemical tags may be the first choice protein-labeling approach. Moreover, the speed and the specificity of the formation of the covalent bond between the HaloTag and the ligand provides new means of protein purification from not easily tractable organisms [48].

Mitochondria are known to be very dynamic organelles undergoing constant antagonist fusion and fission reactions [49]. Several GTPases drive these opposing reactions in a highly regulated manner and the defects in the fusion or fission result in disintegration or collapse of the organelles, respectively. So far no information has been obtained on the machinery controlling the dynamics of mitosomes and hydrogenosomes. Given that neither the components of the mitochondrial division cycle nor the homologues of bacterial division proteins were found in the genomes of mitosome- and hydrogenosome-bearing eukaryotes, the HaloTag has the potential to be a means of identifying the different components driving these processes in these protists.

This opens up more fundamental questions regarding the evolution of the mitochondrial division apparatus, the transition from a FtsZ- to a dynamin-based system as well as the origin of mitochondrial fusion. We believe that the introduction of HaloTag technology to the cell biology of anaerobic protists will be of assistance in the process of answering these questions.

## Supporting Information

Movie S1 *Giardia intestinalis* expressing mitosomal IscU-HaloTag fusion was labeled with TMR-Halo ligand. Images were taken every at 10 second intervals, movie is displayed at 2 frames per second.
(AVI)

Movie S2 Nomarski differential contrast of the same visual field as in Movie S1.
(AVI)

Movie S3 *Trichomonas vaginalis* expressing hydrogenosomal frataxin-HaloTag fusion was labeled with TMR-Halo ligand. Images were taken every at 10 second intervals, movie is displayed at 2 frames per second.
(AVI)

Movie S4 Nomarski differential contrast of the same visual field as in Movie S3.
(AVI)
